# Assessment of immunization data quality of routine reports in Ho municipality of Volta region, Ghana

**DOI:** 10.1186/s12913-020-05865-4

**Published:** 2020-11-04

**Authors:** Sorengmen Amos Ziema, Livingstone Asem

**Affiliations:** 1 Department of Epidemiology, and Biostatistics, School of Public Health, University of Health and Allied Sciences, Ho, Volta Region Ghana; 2 Department of Public Administration and Health Services Management, Business School, University of Ghana, Legon, Accra, Ghana

**Keywords:** Immunization data, Data quality, Expanded program on immunization

## Abstract

**Background:**

Immunization has been an important public health intervention for preventing and reducing child morbidity and mortality over the years and coverage has increased in the past decades. However, the validity of the data from immunization coverages is usually disputed. Immunization data from health facilities show poor concordance between tallied registers and monthly reports as they are reported to higher levels of the health system. The study assessed the quality of data from routine immunization of some health facilities in the Ho central municipality in the Volta region of Ghana.

**Methods:**

A descriptive cross-sectional study was used to review routine immunization data in tallied registers and reports submitted to the Municipal Health Directorate (MHD) from January to December, 2015. Simple random sampling was used to select three health facilities in Ho central municipality. The World Health Organization (WHO) Data Quality Self-assessment (DQS) tool was the main instrument used to present and analyze data for accuracy and discrepancy level between the tallied registers and reports. A template was created in Microsoft excel which automatically presented accuracy and discrepancy levels when data was entered. Ethical approval for the study was obtained from Ghana Health Service Ethics Review Committee.

**Results:**

The result showed discrepancies between recounted tallies at the facilities and reports submitted to the MHD. Accuracy ratios of 102, 64 and 94% for Bacillus Calmette Guerin (BCG), Pentavalent (Penta) vaccine dose 3 and Measles 2 respectively indicating underreporting for BCG and over reporting for the rest were obtained. There was 460 over reported data to the municipal level representing accuracy ratio of 80% and discrepancy level of 20%.

**Conclusions:**

Immunization data was characterized by underreporting and overreporting, hence not accurate and lacked quality. Immunization data quality should be a priority among health staff at health facilities.

## Background

Immunization ensures immunity against various diseases. Acquired immunity is attained through either passive or active immunization. Passive immunization refers to the transfer of active humoral immunity in the form of ready-made antibodies from one individual to another [1, 2]. It can occur naturally by transplacental transfer of maternal antibodies or induce artificially by injecting a recipient with exogenous antibodies targeted to a specific pathogen or toxin [2, 3]. Artificial immunization is used when there is a high risk of infection and insufficient time for the body to develop its own immune response. Active immunization which produces antibodies against a specific agent after exposure to the antigen can be acquired through either natural infection with a microbe or administration of a vaccine comprising attenuated (weakened) pathogens or inactivated organisms [2, 4]. Active artificial immunization is provided in most countries through routine immunization or Expanded Program on immunization (EPI) and as part of primary health care approach [5]. The global effort to use vaccination as a public health intervention began in 1974 when WHO launched the EPI [6, 7]. Most countries since then have made significant efforts in immunization activities ensuring that children are protected from vaccine preventable diseases.

Ghana launched the EPI in June 1978 with six antigens – BCG, measles, diphtheria-pertussis-tetanus (DPT) and oral polio for children under 1 year of age [8]. This was intended to reduce morbidity and mortality of vaccine preventable diseases which then contributed significantly to both infant and child mortality in the country. The EPI was a government policy to ensure all children receive these vaccines before their first birthday. The number of vaccines given to children under 5 years in Ghana at the time of study was 12 [9]. To achieve high coverages, annual targets for both at the district and national levels are set so that health personnel work immensely to reduce vaccine preventable diseases burden among children. Various strategies are used to deliver immunization in many countries. These include static vaccination posts, outreach services as well as campaigns or national immunization days [10]. All these methods of delivery aim to reach out to most of the unreached populations and achieve high coverage so that vaccine preventable diseases are reduced.

After immunization, data is generated through recording the number of children immunized and vaccines used as part of administrative monitoring. Flow of immunization data begins at the health facilities where vaccines are administered [5]. Vaccination is conducted by health personnel in these facilities as either static or outreach services in catchment communities. Typically, when a health worker administers a dose of vaccine, the date of vaccination is immediately recorded on the child’s individual vaccination card and on the immunization register and the dose is tallied on an appropriate sheet allowing for the easy re-counting of all doses provided. The registers and tally sheets are kept in these facilities where the vaccinations are performed. These health facilities usually report these immunization data to the district/municipal health directorates on regular basis (monthly or quarterly) [5]. At the district level, health personnel receive the reports and check for completeness, timeliness, accuracy and follow up on late, incomplete, inaccurate reports. All reports from facilities within the district’s jurisdiction are aggregated and a report sent to the regional level. The region as well collates all districts reports and send to the national level [5].

Immunization coverage over the past decades has increased considerably in most countries [11]. This can be attributed to the commitment by most countries to meeting the then Millennium Development Goal (MDG) 4 of reducing under-5 mortality rate by two thirds of the 1990 levels by 2015 of which immunization was vital. Estimates from WHO indicate that immunization currently prevents 2–3 million deaths every year [12]. Substantial investments continue to be given by international agencies like Global Alliance for Vaccines and Immunizations (GAVI) and WHO to improve immunization coverage in developing countries [11, 13]. However, the quality of data generated by these countries continue to be in contention. Data are usually overreported or underreported from one level of the health system to another [5, 14, 15].

A study in forty-one low income countries on accuracy and quality of immunization information systems found almost half of the countries obtaining a verification factor which measured accuracy of the reporting system below 80% [16]. In their findings on the validity of reported vaccination coverage in 45 countries [17], officially reported diphtheria tetanus-pertussis vaccine (DTP3) coverage was higher than what was reported from household surveys. Immunization information system assessment [18] revealed that, in Kenya, concordance of immunization data between facility monthly report and facility vaccination tally sheets was 31% and 38% in Ghana. A study in Mozambique [5] reported that, numbers of all vaccine types were different when tally sheets, facility registers and district reports were compared. In Ghana, [19] made the case that there were discrepancies between tallied data at the vaccination delivery sites and reported data to the MHD. The integrity and quality of our routine administrative data due to inconsistencies, inaccuracies, errors in our data reporting have always been an issue. The Ho municipality has seen persistent drops of immunization coverage from 2013 to 2015 and one of the worst performers in the country [20]. There is therefore a tendency by lower level health facilities to overreport immunization data to evade continuous reprimands by high level staff. Also, though data verification takes place at the facility level on regular basis, supervisors concentrate more on consistency checks between data in the facility reports and the number of vaccines received. The problem of immunization data inaccuracies between facility registers and reports submitted to higher levels of the health system is widespread and not different in Ghana. From our literature search, limited studies on data quality assessment in the Volta Region of Ghana were found. Hence, the study sought to assess the quality of routine immunization data for 2015 generated in Ho central.

## Methods

### Study setting

The study was conducted from February to March, 2017 in Ho central within the Municipality of the Volta Region of Ghana. The Ho central is one of the four sub-municipals in the Ho municipality comprising of twelve health facilities with the MHD responsible for management and providing support to the sub-municipals. Three of these facilities are hospitals; the regional, municipal and a private hospital and the rest clinics, Community-based Health Planning and Services (CHPS) and family health units. These facilities are manned by trained and skilled health personnel who manage all kinds of health-related issues. Community health nurses at the various Reproductive and Child Health units perform vaccinations in the facilities and during outreach services. Data are generated at the end of vaccination, recorded in assigned books and safely kept. The availability of skilled personnel therefore affects data quality. The study was specifically conducted in two clinics and a CHPS compound located within the central municipality.

### Study population and design

According to the 2017 projected populated by the Ghana Statistical Service, the population of Ho municipality was 209,161 with under-five being 23,734 [21]. Immunization data on under-five children from sampled health facilities were studied. The study was descriptive cross-sectional. This design was chosen because it was appropriate in terms of time for the study. Data of immunized children under 18 months were studied. They were examined in tally books and reports and recorded into DQS tool [18] for analysis.

### Sampling of vaccines and health facilities

Selection of vaccines and study facilities began at the MHD. The three vaccines - BCG, Penta 3 and Measles 2 were selected randomly. This was done using simple random sampling where names of the twelve vaccines were written on pieces of paper and picked without replacement. Health facilities in Ho central that performed immunization and had complete data on the selected vaccines for January to December, 2015 were included. All the hospitals were excluded because they did not vaccinate against Measles 2. Any facility that had zero records of BCG, Penta 3 and Measles 2 were also excluded. Three of the nine eligible facilities were selected using simple random sampling. Selection of three health facilities was based on previous similar study [14]. This was done by writing their names on pieces of paper and picking at random without replacement. Letters A, B and C were used to represent the facilities in the analysis for the purposes of anonymity.

### Definition of terms

#### Data accuracy

The degree to which data has attributes that correctly represent the true value of the intended attribute of a concept or event in a specific context of use [22].

#### Data consistency

The degree to which data has attributes that are free from contradiction and are coherent with other data in a specific context of use [22].

#### Discrepancy

The data from two or more sources are not consistent.

### Measurement of data accuracy and discrepancy

The DQS tool is a standard tool developed by the WHO and use to determine accuracy of immunization data. It is simple to use in comparing immunization data from a source such as tally sheets data to reports submitted to health directorate by same facility and period. This tool has been used in previous studies on data quality and findings established inconsistency between source documents such as tally books and reports generated from same data.

Accuracy ratio is obtained by dividing tallied figure by reported figure all multiplied by 100%. The discrepancy level is obtained by subtracting the accuracy ratio from 100. An accuracy ratio less than 100% indicates overreporting whereas underreporting occurs when accuracy ratio is greater than 100% [23]. Overreporting gives an accuracy figure of less than 100% and positive discrepancy level indicating more data being reported to the MHD than found in the tally registers. Underreporting gives an accuracy figure more than 100% and a negative discrepancy level demonstrating less data being reported to the MHD than recorded in the tally registers.

### Data collection procedure

Reported data from the sampled facilities on BCG, Penta 3 and Measles 2 were first obtained from the MHD. Data on reports were read, recorded and reread for each month per vaccine to avoid errors. Visits were then made to facilities selected and data in tally sheets which are the original source documents were recounted. Tallies on these vaccines were recounted thrice for each month per vaccine at all facilities. The WHO DQS tool [23] was adopted and modified in excel. The data were first recorded in a note book and then transferred into the tool in excel sheet for analysis. In all three facilities involved, visits were made twice because some of the tally books were not available on the first visit.

### Data analysis

Data collected from the different levels were entered into Microsoft excel for storage and analysis. Data upon entry into excel were cross-checked thrice to avoid transcription error. A template of the WHO DQS tool was created in excel. Data for each month per vaccine and for each facility was then entered into the tool to generate accuracy ratio and discrepancy level. Descriptive statistics was done with excel and presented in simple graphs and tables.

### Ethical issues

Ethical clearance for the study was obtained from Ghana Health Service Ethical Review Committee (GHS-ERC) with number GHS-ERC: 64/10/2016. Permission was sought from the MHD through an introductory letter. GHS data request form at the MHD was filled before accessing reports. Letters were rewritten by the Municipal Health Director and sent to the facility in-charges who gave approval before data were accessed. Letters A, B and C were used to represent the 3 facilities for the sake of anonymity.

## Results

Table 1 illustrates the number of children who received immunizations in facility A from January to December 2015 based on the recounted data in tally sheets and submitted reports to the MHD. The recounted tally sheet data for BCG was 345 and 335 was reported figure to the directorate, indicating an underreported number of 10. Again, there was a marginal difference of 4 between tally sheets (367) and the number of submitted reports 363 for Penta 3, representing underreporting for the year 2015 under study. Recounted Measles 2 tallies were 292 as against 307 for the submitted reports, indicating an overreported figure of 15 to the MHD.

**Table 1:** Comparison of recounted tallies and submitted reports in 2015 for facility A

Facility A													
Vaccine/Sources	Jan	Feb	Mar	Apr	May	Jun	Jul	Aug	Sep	Oct	Nov	Dec	Total
***BCG***													
Tally sheet	17	0	47	34	26	31	9	0	91	35	21	34	345
Submitted report	17	0	37	34	26	31	9	0	91	35	21	34	335
***Penta 3***													
Tally sheet	37	19	41	25	41	28	26	28	34	36	24	28	367
Submitted report	38	22	40	25	36	30	26	28	30	34	26	28	363
***Measles 2***													
Tally sheet	30	33	31	19	13	33	21	29	19	24	26	14	292
Submitted report	30	33	31	22	13	33	26	29	19	26	26	19	307

In facility B, there was an overreported figure of 3 between tally sheet and submitted report for BCG as shown in Table 2. Also, recounted tallies for Penta 3 in facility B was 193 as against 201 for submitted reports, showing an overreported figure of 8 to the MHD. Measles 2 recounted tallies was 165 and that of submitted reports was 149 representing underreporting to the health directorate. Similarly, 184 tallies were recounted whereas 599 was reported for Penta 3 in facility C. Same facility for Measles 2 had 282 as recounted tallies and 331 for reports.

**Table 2:** Comparison of tally sheet data and submitted reports in 2015 for facility B

***Facility/vaccine***													
Vaccine/Sources	Jan	Feb	Mar	Apr	May	Jun	Jul	Aug	Sep	Oct	Nov	Dec	Total
***FACILITY B***													
***BCG***													
Tally sheet	0	1	0	2	1	0	2	0	0	4	4	2	16
Submitted report	0	1	0	2	2	1	2	0	0	5	4	2	19
***Penta 3***													
Tally sheet	7	10	20	18	20	9	10	24	23	18	15	19	193
Submitted report	7	10	20	17	20	11	17	20	28	18	13	20	201
***Measles 2***													
Tally sheet	16	16	11	8	16	6	17	8	11	19	16	21	165
Submitted report	13	16	11	8	10	5	10	8	15	16	16	21	149
***FACILITY C***													
***Penta 3***													
Tally sheet	17	12	19	25	10	12	20	25	19	18	7	–	184
Submitted	33	31	48	62	44	36	52	67	52	66	63	45	599
***Measles 2***													
Tally sheet	25	21	25	36	23	23	36	20	31	32	11	–	282
Submitted	25	22	25	36	24	22	36	20	31	33	25	31	331

Table 3 indicates that, 361 tallies were recounted but 354 was the sum of submitted reports for BCG in two facilities. The recounted tallies for Penta 3 was 744 against 1163 in the submitted reports for all facilities. A total of 739 tallies were recounted and 787 was reported to the MHD for Measles 2.

**Table 3:** Comparison of overall data accuracy in the three health facilities

Vaccine	Facilities			
***BCG***	A	B	C	Total
Tally sheet	345	16	–	361
Submitted report	335	19	–	354
Accuracy ratio (%)	103	84	–	102
Discrepancy level (%)	−3	16	–	−2
***Penta 3***				
Tally sheet	367	193	184	744
Submitted report	363	201	599	1163
Accuracy ratio (%)	101	96	31	64
Discrepancy level (%)	−1	4	69	36
***Measles 2***				
Tally sheet	292	165	282	739
Submitted report	307	149	331	787
Accuracy ratio (%)	95	111	85	94
Discrepancy level (%)	5	−11	15	6

**Fig. 1:**
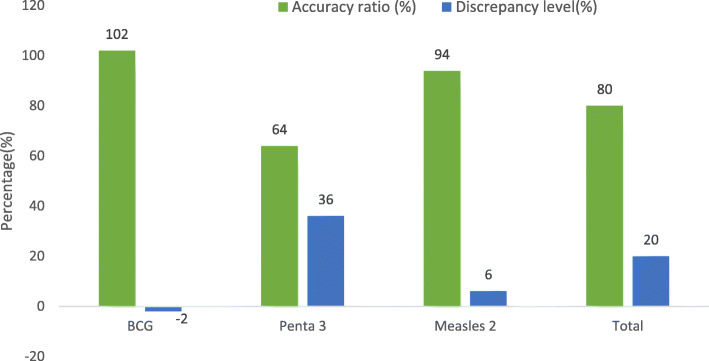
Overall accuracy ratio and discrepancy level for all vaccines and facilities

The overall accuracy ratio for BCG in facilities A and B was 102% with −2% being the discrepancy level, indicating underreporting as presented in Fig. 1. Penta 3 had an accuracy ratio of 64% with 36% discrepancy level for all facilities representing overreporting. Similar overreporting was observed for Measles 2 as 95% accuracy ratio was recorded with 6% discrepancy level for all facilities. For all vaccines and in all facilities, 80% accuracy was witnessed with 20% discrepancy level.

## Discussion

The results revealed that no facility recorded 100% accuracy ratio between the two sources of data for any of the 3 vaccines. An underreporting was observed for BCG data in two of the facilities. This contradicted with the findings of [5] which reported that facilities were overreporting for all vaccine types and the average overreporting was 44% for BCG. Also, [14] stated in their findings that, there was overreporting of all vaccines to the health directorate and 15% for BCG. This underreporting of BCG could be attributed to transcription errors. This is rather bizarre since there is tendency to overreport than underreport to ensure high coverages. When the discrepancy level is more than 10%, then the data is not reliable for decision making and planning of immunization programme [19]. Penta 3 data was highly overreported to the MHD. This agrees with the findings of [5, 14, 19, 24] in their studies where Penta 3 or 3rd dose of diphtheria-tetanus-pertussis witnessed overreporting to higher levels. Penta 3 data for 2015 in the facilities studied was appalling and should be of much concern. This high discrepancy could not have been linked to transcription errors alone. Possibly, some facilities may not have submitted all the tally books for assessment since records keeping at the lower levels is usually problematic. For Measles 2 data, overreporting occurred similar to [19] findings. Reasons for this discrepancy could be attributed to arithmetic and transcription errors by health personnel responsible.

About 460 inconsistent data were found between the two sources [19]. found similar but high numbers, where an average of 668 data per antigen and total of over 2500 figures were over reported to the MHD in each of 2011 and 2012 for the eight antigens assessed [14]. in their study also reported that, the average difference of all vaccines between immunization register and Primary Health Care report was 51.3% depicting overreporting. This study found 80% accuracy and 20% discrepancy level which indicated overreporting to the MHD [18]. assessed Ghana’s information system and found that concordance between facility monthly report and facility vaccination tally sheets was 38%. This confirms the finding of this study that, inconsistency existed between the two sources of data assessed. Reasons attributable to this discrepancy could be arithmetic errors during monthly data compilation, unavailability of all tally sheets data at time of study or deliberate over reporting to achieve high coverage to avoid query by higher levels staff. Arithmetic errors occur when health workers have to add all tallies from vaccination at the end of every month and report to the MHD. Health workers due to busy schedules usually collate these tallies in a haste to meet timelines thereby leading to errors. There is a possibility of tallying vaccination data in papers or other books apart from the recognized registers in times of shortage which might get lost during collation at the end of the month. The demand for high coverage by municipal, regional and national levels without considering local conditions could also contribute to intentional overreporting by facility levels staff. The Ho central witnessed low vaccination coverage in the region, hence the tendency of health workers overreporting to the MHD. As indicated earlier, on the whole, this data was ineligible for planning and decision making since it exceeded the 10% limit [19]. These problems should be addressed by the necessary authorities if immunization data quality in the municipality is to be achieved.

## Limitation

Health facility C could not provide data for BCG. If included could have affected the overall data accuracy ratio despite its exclusion from the analysis.

## Conclusions

Data in source documents (tally books) were inconsistent with submitted reports at the MHD and meant they were inaccurate. This study found that immunization data were characterized by underreporting and overreporting, hence lacking quality. Since much investment is put into vaccinations, quality data in terms of consistency and accuracy should be ensured at all levels and time for evidence-based decision-making to support successful national immunization programmes. Data audit teams should be instituted at all facilities supervised by directorate staff to validate monthly data before submission to the next level. Data quality assessment should be part of the routine monitoring and support visits to all health facilities by municipal/district health directorate staff.

## Data Availability

Available upon request from the corresponding author.

## Abbreviations

BCG: Bacillus Calmette Guerin
CHPS: Community-based Health Planning and Services
DPT: Diphtheria-Pertussis-Tetanus
DQS: Data Quality Self-assessment
EPI: Expanded Programme on Immunization
GAVI: Global Alliance for Vaccines and Immunizations
GHS: Ghana Health Service
GHS-ERC: Ghana Health Service Ethical Review Committee
MHD: Municipal Health Directorate
MDG: Millennium Development Goals
Penta 3: Pentavalent Vaccine dose 3
WHO: World Health Organization
